# Feasibility of high dose dobutamine stress and scar imaging in high field open MRI in patients with suspected coronary artery disease

**DOI:** 10.1186/1532-429X-14-S1-P15

**Published:** 2012-02-01

**Authors:** Philip G Petry, Bernhard Schnackenburg, Hugo A Katus, Henning Steen

**Affiliations:** 1 Facharztpraxis für Diagnostische Radiologie Philip G. Petry, Heidelberg, Germany; 2 Philips Medical Systems, Hamburg, Germany; 3 Department of Cardiology, University of Heidelberg, Heidelberg, Germany

## Background

High-dose dobutamine-stress magnetic resonance (DSMR) and late gadolinium enhanced cardiac magnetic resonance (LGE-CMR) imaging are well established for the detection of significant cardiovascular disease (CAD) and infarction for cylindrical 1.5 T or 3T. Due to the narrow bore, patients (pts) with obesity or claustrophobia usually cannot be studied with conventional MRI scanners. 1.0T high field open (HFO) is a non-cylindrical MRI system offering significantly more space but has only rarely been utilized for diagnostic cardiac MRI so far.

In this pilot study we sought to investigate the performance of 1.0T HFO DSMR and LGE-CMR in pts with suspected CAD and low to intermediate risk profile.

## Methods

We studied 44 pts (29men;15women,55±6ys.) with low to intermediate PROCAM risk-score for significant CAD who couldn’t be scanned on 1.5T due to obesity (24 pts) or claustrophobia (20pts, resistant even after sedation) in a 1.0T HFO (Philips Healthcare, Best, The Netherlands). Dobutamine was administered using established standard protocols, atropine was given to reach heart-rate target if necessary. We employed a standard ECG-and or pulse-gated SSFP-sequence (TR/TE: 4.7/2.2 msec,flip-angle:70°, resolution:1.8×2×8mm3,30 heart phases) for DSMR and a double inversion-recovery sequence (TR/TE:3.9/1.3 msec,flip-angle:15°,voxel:1.7×1.9×5 mm^3^,TFE factor=21) for LGE-CMR. Two-, three- , four-chamber and three short axes image planes were taken step-wise at rest and stress. After stress imaging, LGE CMR was performed to detect potential small myocardial infarctions.

## Results

DSMR and LGE-CMR were successfully performed in all pts. Average stress heart rate was 92±6% of age-predicted maximum heart rate. Eight pts revealed ischemic wall motion abnormalities, which could all be verified as significant CAD by coronary angiography. Sedation could be avoided in 90% of all claustrophobic pts. Mean body-mass-index was 32±4. In 16% of pts, the vector ECG-signal became insufficient under stress at heart rates >120/min but additional peripheral pulse-gating was sufficient in all cases.

## Conclusions

This is the first human DSMR and LGE-CMR study showing that 1.0T HFO is a feasible, accurate and sedation-saving method in obese or claustrophobic pts to enable diagnostic MR stress and scar imaging in this usually not accessible patient collective. Due to the increasing number of obese pts, 1.0T HFO could represent a valuable alternative for conventional narrow bore CMR machines. Although vector-ECG was lost in approximately one sixth of pts, potentially due to the pronounced magneto-hydro-dynamic effect in vertical magnetic fields, pulse-gating was sufficient to terminate all DSMR and LGE-CMR scans successfully.

## Funding

None.

